# Laryngo-tracheal stenosis in a woman with ablepharon macrostomia syndrome

**DOI:** 10.1186/s12890-019-0921-8

**Published:** 2019-08-28

**Authors:** Paola Ciriaco, Angelo Carretta, Giampiero Negri

**Affiliations:** Department of Thoracic Surgery, Scientific Institute and University Vita-Salute San Raffaele, Ospedale San Raffaele, Via Olgettina 60, 20132 Milan, Italy

**Keywords:** Ablepharon macrostomia syndrome, Laryngo-tracheal stenosis, Dyspnea

## Abstract

**Background:**

Ablepharon macrostomia syndrome (AMS) is a rare congenital malformation disorder caused by the autosomal-dominant mutations in gene TWIST2. Patients affected by the disease present abnormalities in ectoderm-derived structures mainly consisting in major facial dysmorphic features and rarely in visceral anomalies. The only laryngo-tracheal defect reported is malacia, with no reference to any anatomical stenosis. We describe a unique case of laryngo-tracheal stenosis in a woman, with genetically confirmed AMS currently followed at our Department.

**Case presentation:**

A 37-year-old Caucasian woman was admitted to the intensive care unit for acute dyspnea that required orotracheal intubation followed by tracheostomy.

The bronchoscopy revealed abnormal tracheal tissue at the level of the cricoid and the first three tracheal rings reducing airway caliber by 80% (grade III according to the Cotton-Meyer classification). Treatment of the stenosis by means of temporary tracheostomy and corticosteroids therapy resulted in airway patency restoration and patient’s return to her normal activities. Bronchoscopy at four and five months showed disappearance of the abnormal tissue and a residual anatomical laryngo-tracheal stenosis of about 20% (grade I according to the Cotton-Meyer classification) of the normal airway caliber.

**Conclusions:**

To our knowledge, this is the first patient affected by AMS presenting with laryngo-tracheal stenosis.

## Background

Typical congenital malformations in ectoderm-derived structures, mainly in the face, characterize ablepharon macrostomia syndrome (AMS). They include: absent or hypoplastic eyelids, fusion defect of the mouth, abnormal ears, skin defects, normal intellectual and motor development, variable abnormalities of the nipples, genitalia, fingers, and hands, and poor growth [1]. AMS was first described in 1977 [2] and since then only few cases have been reported in the literature. Visceral involvement is rare so as airway abnormalities. Anecdotal laryngo-tracheal malacia is present in literature [3–5]. We report a case of laryngo-tracheal stenosis in a woman with genetically confirmed AMS, referred to our Department. The medical history and follow-up are peculiar to a rare case never reported before.

## Case presentation

A 37yo Caucasian woman was seen in May 2018 at our Department of Thoracic Surgery for major dyspnea on exertion. She was known for AMS with the specific genetic mutation in the gene TWIST2 (c.223G > A), no pathogenic sequence changes were found in both her parents [6]. The patient was delivered full term, after an uncomplicated pregnancy. Weight birth and height were 2.97 kg and 49 cm, respectively. Her facial features at birth were hyploplastic eyelids, dysmorphic nose and ears, fusion defect of the mouth, dry and coarse skin, cranial hypoplasia with sparse hair and growth delay. The patient had spent the first three weeks in an incubator for having inhaled the amniotic fluid and meconium. Her intellectual and motor development was normal.

The patient underwent 17 previously maxillofacial operations requiring intubation, including multiple surgeries for eyelids and ears lobes reconstruction, mastoplasty for mammary glands absence and lips correction. The last procedure had been performed ten years before the onset of symptoms. In her lifetime, the patient never complained dyspnea and/or shortness of breath, although she reported a quiet lifestyle.

At the time of our evaluation, she reported dyspnea on exertion in the previous 18 months with an acute episode that occurred six months before. The acute dyspnea episode caused intensive care unit (ICU) admission, with orotracheal intubation by means of a 5 mm tube, subsequent tracheostomy and prolonged mechanical ventilation. Flexible ICU bronchoscopy showed endotracheal tissue approximately at the cricoid level and the first three tracheal rings resulting in an airway stenosis of 80% (grade III according to the Cotton-Meyer classification), compatible with inflammatory edema of the mucosa, above the tracheostomy site. The distal trachea was normal.

She received corticosteroid obtaining a resolution of symptoms and a return to spontaneous breathing. Tracheostomy cannula was removed after 19 days and the patient was discharged from the hospital.

**Fig. 1:**
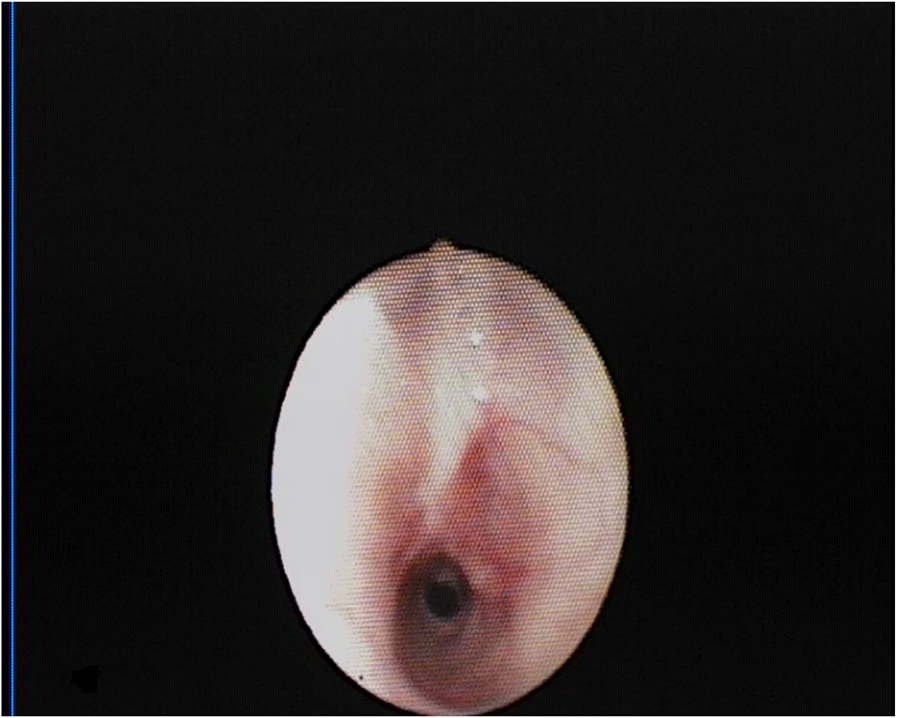
Endoscopic view of cricoid and trachea June 2018, showing laryngo-tracheal stenosis reducing the airway caliber by 50%

**Fig. 2:**
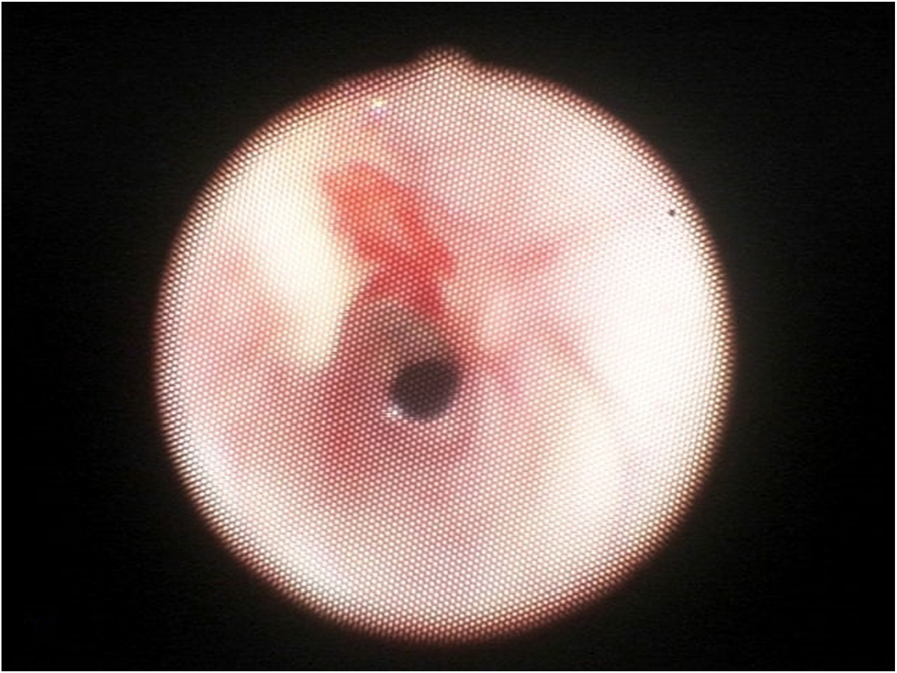
Endoscopic view of cricoid and trachea at 40 days, July 2018 showing improvement of laryngo-tracheal stenosis reducing now the airway caliber by 20%

**Fig. 3:**
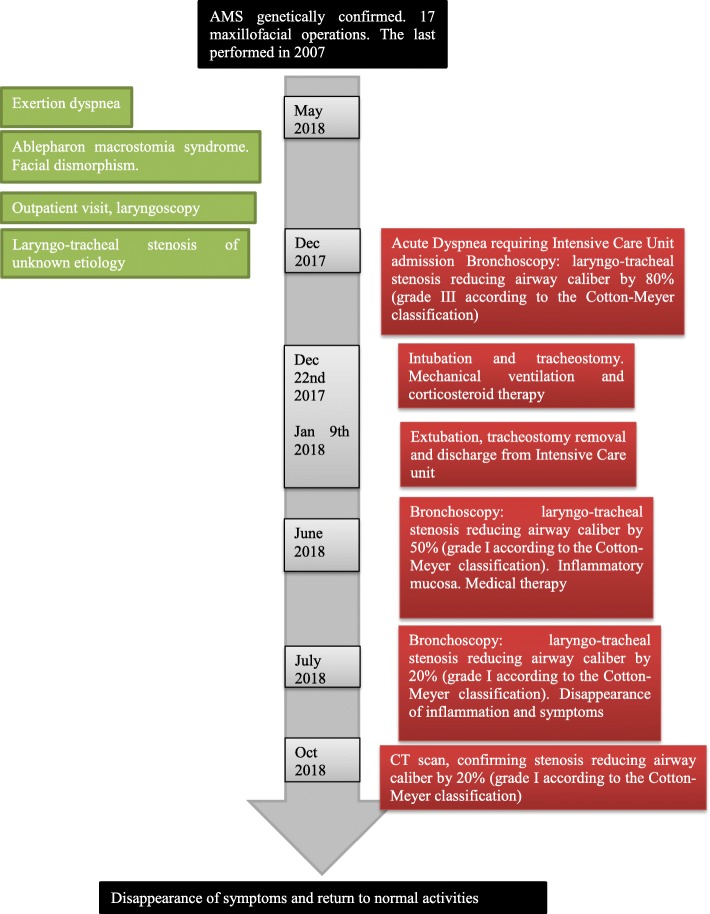
Timeline

The patient returned to her normal daily activities. Thereafter, she continued to complain of dyspnea for intense efforts, despite the improvement in the symptoms. She was then referred to our Department for reevaluation. Laryngoscopy showed a normal glottis. A flexible bronchoscopy performed at the beginning of June 2018 revealed a circumferential airway stenosis involving the cricoid cartilage and the first tracheal ring, resulting in an airway stenosis of 50% (grade I according to the Cotton-Meyer classification) (Fig. 1), with acute inflammation of the mucosa. There was no laryngo-tracheal malacia. The size of the airway was adequate for ventilation. The dilation of the stenosis was not carried out immediately due to the presence of acute inflammation of the mucosa and sub-critical reduction of airway diameter. Symptoms improved significantly after corticosteroids therapy. Endoscopic follow-up was performed at 40 days showing amelioration of the mucosa’s inflammation although a laryngo-tracheal stenosis was still present, resulting in an airway stenosis of 20% with disappearance of inflammatory signs (grade I according to the Cotton-Meyer classification) (Fig. 2). In view of the improvement in symptoms and stenosis, no other maneuvers have been scheduled except for bronchoscopy after three months. This bronchoscopy was not performed, due to lack of symptoms. A computed tomography scan, instead, confirmed a grade I laryngo-tracheal stenosis according to the Cotton-Meyer classification (Fig. 3).

## Discussion and conclusions

AMS is an infrequently reported congenital malformation disorder caused by mutations in the TWIST2 gene causing defects in ectoderm-derived structures [1]. Abnormalities are mainly facial and rarely visceral organs are involved. Less than 20 cases of AMS are reported in the literature and to our knowledge, none of them presented with airway stenosis [1, 3, 4, 7, 8]. The patient observed in our Department underwent numerous orotracheal intubations due to previous maxillofacial surgery. The onset of respiratory symptoms occurred ten years after the last operation, with no previous symptoms. In addition, no history of difficult intubation was reported. A definite etiology of the laryngo-tracheal stenosis could not therefore be established. It is possible that an acute inflammatory process had worsened a previous unknown stenosis of the airway and corticosteroids therapy contributed to symptom resolution. The computed tomography scan performed at three months, confirmed the presence of tracheal stenosis. Temporary tracheostomy was helpful to allow mechanical ventilation before the resolution of symptoms. In addition, medical therapy helped the resolution of symptoms and restoration to spontaneous breathing.

Laryngo-tracheal anomalies do not belong to the AMS phenotype [9] but might be present in 40% of the case of Fraser syndrome [9, 10]. The two syndromes are reported to overlap clinically each other although in AMS no mutation in the responsible genes for Fraser syndrome (FRAS1, FREM2 or GRIP1) was found. In our patient differential diagnosis was possible considering her negative FRAS1 mutational screening.

In light of the patient’s history, we hypothesize that the laryngo-tracheal stenosis has been always present in this woman, although primary etiology cannot still be recognized. To our knowledge, this is the first case of laryngo-tracheal stenosis in AMS reported in the literature. Further considerations are necessary after future patient evaluation.

## Data Availability

Availability data and material is not applicable since this is a case report. Significant pictures related to the paper are included in the case report. Other data owned by the Authors are confidential/identifying patient data (face pictures, genetic data).

## Abbreviation

AMS: Ablepharon macrostomia syndrome
